# Effects of multimodal agility-like exercise training compared to inactive controls and alternative training on physical performance in older adults: a systematic review and meta-analysis

**DOI:** 10.1186/s11556-021-00256-y

**Published:** 2021-02-25

**Authors:** Mareike Morat, Tobias Morat, Wiebren Zijlstra, Lars Donath

**Affiliations:** 1 Institute of Exercise Training and Sport Informatics, Department of Intervention Research in Exercise Training, German Sport University Cologne, Am Sportpark Muengersdorf 6, 50933 Cologne, Germany; 2 Institute of Movement and Sport Gerontology, German Sport University Cologne, Am Sportpark Muengersdorf 6, 50933 Cologne, Germany

**Keywords:** Agility-like training, Agility inspired training, Multimodal exercise training, Meta-analysis

## Abstract

**Background:**

Multimodal exercise training (MT) as a time-efficient training modality promotes a wide range of physical dimensions. Incorporating agility-like training aspects (coordination, changes of direction and velocity) into MT may further enhance physical outcomes highly relevant for activities of daily living. This meta-analysis investigated the effects of multimodal agility-like exercise training (MAT) on physical and cognitive performance compared to inactive (IC) and active controls (AC) in older adults.

**Methods:**

Literature search was conducted in four health-related databases (PubMed, SCOPUS, SPORTDiscus and Web of Science). Randomized controlled trials with pre-post testing applying MAT (including aspects of training with at least two different traditional domains: strength, balance, endurance) and an agility-like component in community-dwelling older adults were screened for eligibility. Standardized mean differences (SMD) adjusting for small sample sizes (hedges’ g) were used to extract main outcomes (strength, gait, balance, mobility, endurance, cognition). Statistical analysis was conducted using a random effects inverse-variance model.

**Results:**

Twenty trials with 1632 older adults were included. All effects were significantly in favour of MAT compared to IC: Strength, mobility and endurance revealed large overall effects (SMD: 0.88, 0.84, 1.82). Balance showed moderate effects (SMD: 0.6). Small overall effects were observed for gait (SMD: 0.41). Few data were available to compare MAT vs. AC with negligible or small effects in favour of MAT. Funnel plots did not reveal clear funnel shapes, indicating a potential risk of bias.

**Conclusions:**

MAT may serve as a time-efficient training modality to induce positive effects in different physical domains. Compared to isolated training, MAT allows equal effect sizes at lower overall training volumes. More studies are needed to investigate the potential value of MAT with systematic training and load control, especially compared to other exercise-based interventions.

**Supplementary Information:**

The online version contains supplementary material available at 10.1186/s11556-021-00256-y.

## Introduction

The worlds’ population is gradually ageing due to an increasing life expectancy [1]. Living those gained years without or with less disabilities requires a health care system focusing on physical, cognitive, and psychosocial wellbeing from a preventive and multidisciplinary perspective [2]. Regular physical activity can attenuate the risk of multiple ageing related diseases [3]. To improve or maintain adequate physical and cognitive performance, exercise training needs to be challenging and multimodal. Since training-induced adaptations have been reported to be very task- and exercise-specific [4, 5], selectively addressing the many dimensions of physical and cognitive performance does not reflect the integrative character of everyday life situations [6].

Established exercise training guidelines for older adults comprise a variety of separate recommendations for relevant main training domains: endurance, strength, balance, and flexibility training [7]. Highly topical, the recently published “guidelines on physical activity and sedentary behaviour” by the World Health Organization (WHO) also take up these aspects. Besides aerobic physical activity, the WHO proposes muscle-strengthening activities that involve all major muscle groups on at least 2 days a week for additional health benefits and varied multicomponent exercises with focus on functional balance and strength training to enhance functional capacity and to prevent falls [8]. The latest Cochrane-Review on fall prevention [9] showed reductions of the rate of falls and the number of fallers by implementing balance exercises combined with functional exercises and by multi-component exercise interventions including balance, functional and strengthening exercises. This also supports a multimodal exercise training (MT) approach, combining different training domains. Furthermore, an approach combining gait, balance and functional training was already set as one of the categories to classify and describe fall-prevention interventions in the taxonomy for exercise interventions by the Prevention of Falls Network Europe (ProFaNE) in 2011 [10]. MT seems particularly essential, since subjectively perceived “lack of time” is among the most reported barriers for the uptake and maintenance of exercise training programs in (older) adults and adherence decreases with every additional weekly training session [3, 11]. Thus, exercise training needs to be time efficient and should integratively train all relevant physical and cognitive performance domains.

For an integratively promotion of balance, strength, and endurance with functional, progressive exercises, combined start-stop and change of direction movements, Donath and colleagues [12] proposed an agility-based exercise training framework for fall prevention for older adults. Within the framework, complex functional tasks, including perception, decision making, reaction, and changes of direction are considered. Task complexity and difficulty are progressively increased in a supervised, group-based training setting. The underlying exercises can vary by changes of the physical, perceptual, or cognitive demands of each exercise or a combination of exercises. Higher physical and cognitive demands also enable cardiovascular and cognitive training stimuli, respectively [12].

However, the term “agility” is not established across literature with older adults, yet. Some intervention studies that designed their training considering aspects of the agility framework used different wording. Those studies refer to group-based exercise training twice or three times per week including multiple physical training components and agility-like aspects. Improvements in strength [13–15], balance [16, 17], cognition [18] and endurance performance [14, 17] were then reported, whereas the greatest improvements have been found in functional mobility outcomes [14, 15, 19].

Based on our knowledge, the agility-based exercise training framework by Donath et al. [12] was not extensively implemented in fall prevention programmes for older adults, yet, but there are several studies that already included some aspects of this framework with older adults not being at high risk for falls [13–19]. However, to date, no meta-analytical evaluation of the effects of multimodal agility-like exercise training (MAT) in older adults has been conducted.

Against this background, the objectives of this meta-analysis are to calculate and classify the effects of MAT compared to an inactive (IC) and/or active control (AC) condition regarding physical (lower and upper body strength, overall strength, gait, balance, mobility, endurance) and cognitive performance in community-dwelling older adults, to describe the present training characteristics of MAT for older adults and to provide recommendations for future research and exercise training practice.

## Methods

### Protocol and registration

This meta-analysis was conducted according to the PRISMA guidelines [20]. This meta-analysis was registered in PROSPERO: CRD42020157205.

### Search strategy and study selection

Literature search was conducted in four health-related databases (PubMed, SCOPUS, SPORTDiscus and Web of Science) until November 21st, 2020. Boolean conjunctions (OR/AND/NOT) were used to combine relevant search terms (operators). These were applied on three search levels (see Table 1).

**Table 1:** Levels and terms of the literature search process

Search level	Search terms with Boolean operators
Search #1	(intervention OR interventional OR interventions OR training OR exercise OR exercising OR exercises)
Search #2	#1 AND (multimodal training OR multi-modal training OR multi-component training OR multicomponent training OR multimodal intervention OR multi-modal intervention OR multi-component intervention OR multicomponent intervention OR multimodal exercise OR multi-modal exercise OR multi-component exercise OR multicomponent exercise OR multimodal exercises OR multi-modal exercises OR multi-component exercises OR multicomponent exercises OR resistance OR strength OR strengthening OR power OR weight-bearing OR speed OR sprint OR balance OR balancing OR coordination OR coordinative OR posture OR postural OR proprioceptive OR proprioception OR sensorimotor OR sensorimotoric OR sensori-motor OR sensori-motoric OR instability OR perturbed OR perturbation OR perturbations OR cognitive OR cognition OR endurance OR aerob OR aerobic OR cardiologic OR cardio OR cardiovascular OR cardio-vascular OR agile OR agility)
Search #3	#2 AND (senior OR seniors OR elder OR aged OR elderly OR old OR older OR aging OR ageing)
Search #4	#3 NOT ((patients OR patient) NOT disease NOT stroke NOT diabetes NOT neuropathy NOT amputation NOT multiple sclerosis NOT cerebral palsy NOT parkinson NOT neoplasms NOT cancer NOT obesity NOT obese NOT osteoarthritis NOT fractures NOT fracture NOT physiopathology NOT dysfunction NOT cognitively impaired NOT frail NOT demented NOT pilot study NOT rheuma NOT rheumatic NOT rheumatoid NOT dietary NOT supplements NOT dietary supplements NOT supplementation NOT drugs NOT abuse)

Hand searching within primary articles and review articles was additionally carried out. All duplicates were removed, before the remaining studies underwent manual screening on three screening levels: 1) title, 2) abstract and 3) full-text. Two independent researchers (MM, TM) conducted the entire process. Irrelevant articles were excluded according to the following criteria. Both researchers achieved a final consensual decision.

The following inclusion criteria were applied for manual screening of the studies:

- Full-text article from peer-reviewed journals in English language
- Randomized controlled intervention study with pre-post testing
- One or more control group(s), receiving no intervention (= inactive control group, IC) and/or receiving an alternative exercise-based training program (= active control group, AC)
- Participants were community-dwelling older adults
- Participants mean age of 65 years or older
- Multimodal exercise training intervention that included aspects of training with at least two different domains (strength, balance, endurance) and an agility-related component (coordination or change of direction and velocity)
- Exercise intervention lasting for at least 6 weeks with a minimum of two weekly training sessions
- Exercise training in a supervised group setting
- Outcome measures that included at least one of the following domains: strength, gait, mobility, balance, endurance, cognition

The following exclusion criteria were applied for manual screening of the studies:

- Older adults with mental declines, acute and chronic cardiac, orthopaedic and/or neurologic conditions
- Competing (master) athletes
- Hospitalized and/or institutionalized older adults
- Older adults at risk of falling or with a serious fall event that led to medical attention (e.g. broken bones) within 1 year prior to the start of the study
- Intervention including nutritional supplementation
- Technology-based intervention
- Study without a comparison group

### Assessment of methodological quality

Methodology of the included studies was rated using the PEDro scale obtained from the Physiotherapy Evidence Database [21]. It comprises 11 dichotomous items (either yes = 1 or no = 0). However, only ten items will be summed up to the final score. Two researchers (MM, TM) independently rated all included studies and arrived at consensus on every item after completing the individual rating process. Raters were not blinded to the study authors and results.

### Data extraction and synthesis

Two researchers (MM, TM) extracted the following primary or secondary outcome domains:

- overall strength (lower body and upper body strength)
- gait
- balance
- mobility
- endurance
- cognition

For strength, isokinetic, isometric and dynamic strength measurements, jumps and tests for muscular endurance (e.g. push-ups, sit-ups) were considered. Assessments, measuring gait speed over a defined distance were taken into account for the gait domain [10]. Balance measures comprised all assessments that determine balance (different stance positions on firm and uneven surfaces, dynamic walks e.g. tandem walk, the functional reach test or test batteries [e.g. Berg Balance Scale] [10]. Mobility tests were chosen as such tests, that depict activities of daily living and are not solely assignable to one of the other domains such as the Timed Up and Go Test (TUG) as a measure of performance including dynamic balance and mobility and the Sit to Stand Test (STS) examining lower body muscle function [22], figure- of-8 running, maximum step length test and the completion of obstacle courses. The successful performance of the included tests is determined by several factors like the STS, which was shown to represent a particular transfer skill, rather than a proxy measure of lower limb strength influenced by multiple physiological and psychological processes [23]. For endurance, the 6 min walk test and the 2 min step test were involved. Cognition comprises all emerging measures that are entitled as such in the respective articles.

Data were transferred to an excel spreadsheet. Relevant study information such as authors, publication year, study design, sample size, gender and mean age of participants, groups and group size, training characteristics, training design, load control and progression, outcome measures, adverse events, adherence rates were extracted. Groups were considered as IC when participants received no treatment at all and as AC when they received any other treatment as MAT. In two studies, one of two AC had to be chosen for further analysis. Resistance training was selected over balance [14] and over coordination [15] training, because resistance training was part of most of the AC of other relevant studies and thus, the homogeneity of AC was higher.

We grouped all active, exercising control groups (*n* = 247), leaving out the PC training group. In case of missing outcome data, authors were contacted via email and asked to provide relevant means with standard deviations. If no answer were received, the respective results could not be integrated in this meta-analysis. If two published articles were included that clearly originated from the same study with the same sample, both articles were merged and treated as one for data analysis to avoid overrepresentation of study results.

### Statistical analysis

For each study, standardized mean differences (SMD, with 90% confidence intervals [CIs]) were computed separately. Therefore, the difference of the target outcome measure between the intervention and the respective control condition including the pooled standard deviations were computed for each outcome. If one study reported several outcome measures of one domain, effect sizes and standard errors were pooled. An inverse-variance method was computed according to Deeks and Higgins [24]. Analyses were conducted applying a random effects model [25]. Forest plots were built for the respective outcome measure category. The following scale was used to classify the magnitude of SMD: 0–0.19 = negligible effect, 0.20–0.49 = small effect, 0.50–0.79 = moderate effect and 0.80 = large effect [26]. Study heterogeneity was assessed using *I*^2^ [27]. A qualitative funnel plot evaluation was performed to assess the risk of a potential bias [28]. All statistical analyses were computed using the Cochrane Review Manager Software (RevMan 5.3, Cochrane Collaboration, Oxford, UK).

## Results

### Trial flow

**Fig. 1:**
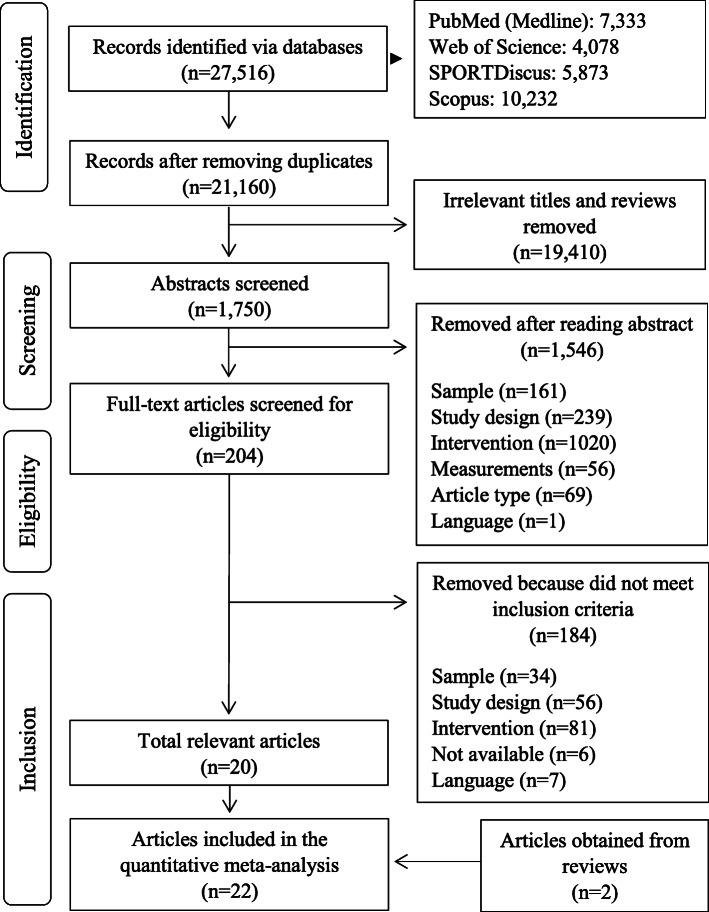
Flow of study screening and selection

Twenty-seven thousand five hundred sixteen potentially relevant articles were found (see Fig. 1). Twenty-one thousand one hundred sixty article titles were cautiously screened for relevance after removing duplicates. One thousand seven hundred fifty potentially relevant articles remained for abstract screening. After thoroughly studying the abstracts, 204 full-texts were further reviewed. One hundred eighty-four did not meet the inclusion or were excluded according to the exclusion criteria. Two additional articles were obtained from reviews leading in total to 22 studies that were finally included in the quantitative meta-analysis. In two cases [29–32], two articles were based on the same study with identical samples. Thus, the two studies were merged respectively and integrated as one study in each case during the further steps of the meta-analysis. They are presented as Ansai et al. [29, 30] and Lord et al. [31, 32] respectively, resulting in a total of 20 studies for analysis. Two other articles also originated from the same study, but different intervention periods were examined, so that the more recent study was selected for analysis [33, 34]. All studies were published 1995 or later. Three studies did not report all relevant data as means ± standard deviations [18, 35, 36]. Authors were contacted and asked to provide missing data. One author [36] answered the request, so that one dataset for balance performance [35] and one dataset for endurance performance [18] had to be left out of further analysis.

### Study population and quality

In the 20 studies, 1632 community-dwellers with a mean age of 72.4 ± 4.3 years were assessed (see Table 2). The sample size ranged from 32 [34] to 259 [18] participants, with a mean size of 82 ± 64 participants. The sample sizes among the studies were not normally distributed (Kolmogorov-Smirnov Test: *p* < 0.001). Trials comprised the following study arms: MAT (*n* = 655), IC (*n* = 570), computer-training group (*n* = 92), fitness intervention (*n* = 108), strength training (*n* = 104), balance training (*n* = 37), coordination training (*n* = 20), strength and balance training (*n* = 19) and strength and endurance training (*n* = 16). Thus, exercise control groups comprised 247 participants as described in the methods section. Accordingly, 1472 participants (655 + 570 + 247; 90%) were finally included into the meta-analysis.

**Table 2:** Overview of the included studies

Reference	Study design	Sample: sample size (male/fe-male); age (M ± SD); drop outs	Groups (group size)	Training character-istics	Training design	Load control and progression	Outcome measures	Adverse events; adherence	Study quality (PEDro)
Ansai et al. 2016 [29, 30]	randomized, three-armed controlled trial	*n* = 69 (22/47); 82.4 ± 2.4 years; *n* = 1	MT: multicomponent training (*n* = 23); RT: resistance training (*n* = 23); CG: no treatment (*n* = 23)	16 weeks; 3 sessions/week; 60 min/session	Warm-up (cycle ergometer); endurance; strength (major muscle groups: upper limb, abdominal, lower limb; with dumbbells, ankle weights); balance/coordination (static balance, static and dynamic weight transfer, walking on a line, walking on unstable surfaces, obstacle transposition and deviation); cool-down (stretching)	Endurance: 60–80% of reserve heart rate adjusted by age and sex; 3x3min; reserve heart rate increased every 3 weeks; strength: 14–17/20 RPE; up to 3 × 15 repetitions	Mobility: TUG with cognitive task, with motor task (time [s]); 5 repetition STS (time [s]); balance: 1-legged standing right, left (time [s]); tandem standing (time [s]); cognition: MoCA (score), CDT (score), verbal fluency task (score)	*n* = 9 (mild muscle pain in MT); 35% of the MT and 57% of the RT training group carried out at least 50% of the sessions	7
Bohrer et al. 2019 [19]	randomized, two-armed controlled trial	*n* = 34 (n.a. /n.a.); 70.3 ± 5.7 years; *n* = 6	EG: multicomponent training (*n* = 16); CG: no treatment (*n* = 18)	12 weeks; 3 sessions/week; 45 min/session	Warm-up (dancing or game); strength; agility (lateral displacements, transpositions of obstacles, hopping); coordination; cool-down	Strength: 12–16/20 RPE; participants were encouraged to perform the concentric phase as fast as they could; 2 min rest between exercises; progression of loads every 2–3 weeks; maintaining movement speed; agility/coordination: increasing difficulty of execution	Strength: isokinetic ankle extension and flexion (peak torque [Nm/kg]); mobility: TUG (time [s]); gait: 10 m walking (speed [m/s])	n.a.; > 80% for all participants	5
Braga et al. 2020 [37]	randomized, two-armed controlled trial	*n* = 32 (n.a./n.a.); 71 ± 2.1 years; *n* = n.a.	EG: multicomponent training (*n* = 16); CG: no treatment (*n* = 16)	12 weeks; 3 sessions/week; 40 min/session	Warm-up (stretching, calisthenics); agility (forward-backward running, direction-changing footwork, climbing/descending stairs); strength (squats, push-ups, sit-ups, jumping jacks, walking lunges, dips, six-point support planks, pulse lunges, reverse lunges, skipping, mountain climbers, arm and leg raises); cool-down	30, 45 or 60s of recovery, always 2x greater than stimulus time, encouragement for passive recovery	Strength: muscular endurance of upper limbs as push-ups (repetitions); muscular endurance of abdomen as sit-ups (repetitions), standing jump (distance), isometric hip, knee and ankle strength (time); endurance: 6 min walking (distance [m])	*n* = 0; n.a.	4
Carvalho et al. 2009 [38]	randomized, two-armed controlled trial	*n* = 57 (0/57); 68.9 ± 3.5 years; *n* = 0	EG: multicomponent training (*n* = 32); CG: no treatment (*n* = 25)	32 weeks; 2 sessions/week; 60 min/session	Warm-up (slow walking, calisthenics, stretching); endurance (slow walking, jogging, dancing, aerobics, step choreographies with steps); strength (stair stepping, knee flexion, arm raise, shoulder abduction, shoulder adduction, shoulder rotation, squatting, biceps curl, triceps extension; with stairs, elastic bands, free weights); agility/reaction (games with change of direction and velocity, response to diverse stimuli; with sticks and balloons); balance (static, dynamic); cool-down (stretching, respiration)	Endurance: 12–14/20 RPE; no progression; strength: 12–16/20 RPE; 1–8 repetitions; increase to up to 2 × 12+ repetitions; agility/reaction: gradually decreased hand support	Strength: arm curl (repetitions [number]); mobility: 30s chair-stand (repetitions [number]), 8-ft up-and-go (time [s]); endurance: 6 min walking (distance [m])	n.a.; 91% in EG (84–100%)	5
Caserotti et al. 2008 [39]	randomized, two-armed controlled trial	*n* = 44 (44/0); 75 ± n.a. years; *n* = 5	TG: multicomponent training (*n* = 16); CG: no treatment (*n* = 28)	36 weeks; 2 sessions/week; 60 min/session	Warm-up; endurance (e.g. walking, running); strength (e.g. half-squat; with elastic tubes); postural control; stretching; reaction	Endurance: 65% of maximal heart rate; at least 30 min; strength: continuous individual increase of repetitions, duration and intensity	Strength: counter movement jump, squat jump (height [cm]); mobility: 5-repeated chair rise (time [s]); gait: 10 m maximal walking, 30 m maximal walking (speed [m/s])	n.a., n.a.	5
Cwirlej-Sozanska et al. 2018 [13]	randomized, two-armed controlled trial	*n* = 44 (8/36); 67.6 ± 3.7 years; *n* = 6	MME: multicomponent training (*n* = 21); CG: health education (*n* = 23)	16 weeks; 2 sessions/week; 60 min/session	Warm-up (low active exercises, breathing); endurance; balance (with and without visual control); strength (e.g. with elastic bands); agility (body position change, gripping, lifting and moving objects, gait re-education); stretching (arms, shoulders, girdle, legs, trunk); cool-down (relaxation, breathing); simultaneous cognitive elements (counting backwards, memorizing and associating certain words with movements of extremities)	Strength: 80% of 1RM; gradual progression of load; endurance: 40–50% of maximal heart rate for 15 min; increase to 50–70% for at least 20 min	Strength: arm curl (repetitions [number]); mobility: TUG (time [s]), 30s chair stand (repetitions [number]), 8-ft up-and-go (time [s]); balance: functional reach (distance [cm]), tandem stance, tandem walk, tandem pivot (scale), static balance (COP sway [mm]); endurance: 2 min stepping (repetitions [number])	*n* = 0; n.a.	6
Englund et al. 2005 [40]	randomized, two-armed controlled trial	*n* = 48 (0/48); 73.0 ± 4.3 years; *n* = 6	EG: multicomponent training (*n* = 24); CG: no treatment (*n* = 24)	48 weeks; 2 sessions/week; 50 min/session	Warm-up; strength (legs, abdominal, back muscles by means of body weight; with dumbbells); endurance/coordination (walking, jogging, steps in different combinations and directions with coordinated arm movements); balance/coordination (one-leg standing and more advanced coordinated steps); cool-down (stretching)	Intensity self-rated; participants allowed to rest if necessary; Strength: 2 × 8–12 repetitions; dumbbell weight increased in progressive phase; Balance/coordination: more complex or faster movements	Strength: isometric knee extension, isometric handgrip strength (force [N]); gait: 30 m maximal walking (speed [m/s]); balance: 1-legged stance (time [s]), Berg Balance Scale (scale)	n.a., 67% (23–95%)	5
Freiberger et al. 2007 [41]	randomized, three-armed controlled trial	*n* = 217 (120/97); 75.9 ± 4 years; *n* = 26	PI: multicomponent training (*n* = 65); FI: fitness intervention (*n* = 69); CG: no treatment (*n* = 83)	16 weeks; 2 sessions/week; 60 min/session	Strength (with dumbbells, ankle weights); balance (standing balance, dynamic weight transfer and stepping strategies); coordination (activities of daily living, activities under time pressure, sensory awareness); perception (including body orientation and space perception); competence training (social competence, material competence, enhancing identity)	n.a.	Mobility: TUG (time [s]), STS (time [s]), maximum step length (distance [cm]); gait: 10 m walking, 10 m maximal walking (speed [m/s])	*n* = 0; PI: median number of sessions attended = 26 (81%, 0–32), FI: median number of sessions attended = 26 (81%, 0–32	7
Karinkanta et al. 2007 [14]	randomized, four-armed controlled trial	*n* = 149 (0/149); 72.6 ± 2.3 years; *n* = 5	RES: resistance training (*n* = 37); BAL: balance-jumping training (*n* = 37); COMB: multicomponent training (*n* = 38); CON: no treatment (n = 37)	48 weeks; 3 sessions/week; 50 min/session	Warm-up; strength (raising from a chair with weight vest, squatting, leg presses, hip abduction, hip extension, calf rise, rowing; with strength training machines); balance/agility (impact exercises, changes of direction, acceleration, deceleration back and forth, sideways walking with stops and turns with music); cool-down	Strength: 2 × 10–15 repetitions at 50–60% of 1RM; 3 × 8–10 repetitions at 75–80% 1RM with > 18/20 RPE; 2 min rest between sets; balance/agility: customization; increase of difficulty of movements, steps, impacts and jumps	Strength: leg press (force [N]); mobility: figure-of-8 running (time [s])	*n* = 14 (musculoskeletal injuries); 67% (74% in RES, 67% in COMB, 59% in BAL)	7
Klusmann et al. 2010 [18]	randomized, three-armed controlled trial	*n* = 259 (0/259); 73.6 ± 4.2 years; *n* = 29	EG: multicomponent training (*n* = 91); PCG: computer education (*n* = 92); CG: no treatment (*n* = 76)	24 weeks; 3 sessions/week; 90 min/session	endurance (bicycle ergometer, treadmill); strength; stretching; balance; coordination	n.a.	Cognition: Rivermead behavioural memory test - immediate recall (score), − delayed recall (score), free and cued selective reminding test - short delay (score), − long delay (score), semantic verbal fluency (score), stroop test (score), trail making test A/B (score); endurance: 6 min walking (distance [m])	n.a.; n.a.	8
Kovacs et al. 2013 [35]	randomized, two-armed controlled trial	*n* = 76 (0/76); 68.4 ± 5.9 years; *n* = 7	EG: multicomponent training (*n* = 38); CG: no treatment (*n* = 38)	25 weeks; 2 sessions/week; 60 min/session	Warm-up (stretching); strength/balance (sitting on a chair: arm lifting overhead, reaching towards floor on side; standing: steps in all directions, reaching overhead, partial squats, turning in standing, tandem standing, sit to stand and stand to sit, high stepping in place; chairs); agility (relay race with heel walking, toe walking, walking on line, walking on exercise mat, slalom around cones, backward walking with second task, ballgames; with balls, cones, exercise mats); cool-down (stretching, breathing)	Strength: 4–8 repetitions; individual adaptation of exercises; other: decreasing support, increasing distances, increasing difficulty, increasing speed; balls with increasing sizes and weights; progressive repetitions and difficulty	Mobility: TUG (score); balance: 1-legged stance (time [s])	n.a., 81% (56–100%)	8
Leite et al. 2015 [42]	randomized, two-armed controlled trial	*n* = 52 (22/30); 69.1 ± 3.2 years; *n* = 9	EG: multicomponent training (*n* = 26); CG: resistance training (*n* = 26)	12 weeks; 2 sessions/week; 75-90 min/session	Warm-up; coordination; strength; agility (integrated cognitive challenges, moving through space using walking; with hurdles, ropes); floor exercises (stretching, strength, relaxation)	Walking faster, longer steps, adding movements of the arms, increasing difficulty; reducing base of support	Strength: isometric handgrip strength (strength [kg]); mobility: chair stand (time [s]); gait: 7 m normal walking, 7 m maximal walking (speed [m/s]); endurance: progressive submaximal exercise test (VO_2_peak [ml/kg/min])	n.a.; EG: 90%, CG: 91%	5
Lord et al. 1996 [31, 32]	randomized, two-armed controlled trial	*n* = 160 (0/160); 71.1 ± 5.2 years; *n* = 28	EG: multicomponent training (*n* = 80); CG: no treatment (*n* = 80)	20 weeks; 2 sessions/week; 60 min/session	Warm-up; endurance (fast walking, stepping, leg lifts, placing foot to the front, side and behind, lunging, heel rises, trunk rotation, flexion, extension of neck, back and pelvis, knee lifts, opposite elbow to raised knee, pelvic floor contractions, belly dancing, shoulder extension, flexion, adduction, abduction, rotation, circling arms, biceps curls, bench press, row, shoulder lever, mock boxing, shoulder rolls, shrugs); strength (lifting bodyweight, e.g. modified push-ups, while seated, lifting leg off floor while resisting movement with hand pressing on knee); balance/coordination (standing on one leg with other raised, ball games requiring catching while standing or moving, kicking a ball, throwing, running under skipping rope, team ball games); stretching; cool-down (relaxation)	n.a.	Strength: isometric knee extension, knee flexion, ankle dorsiflexion, hip extension, hip flexion (force [N]); gait: 11.2 m walking (speed [m/s]); balance: stance on floor - eyes open, − eyes closed, stance on foam - eyes open, − eyes closed (sway [mm])	n.a., 73.2%	4
Marques et al. 2011 [16]	randomized, two-armed controlled trial	*N* = 60 (0/60); 69.9 ± 5.8 years; *n* = 11	EG: multicomponent training (*n* = 30); CG: no treatment (*n* = 30)	32 weeks; 2 sessions/week; 60 min/session	warm-up (stretching); strength (marching in place, heel-drops, stepping, squats, hip flexors, extensors, abductors, knee flexors and extensors upper body exercises; with weight vests, stepper, elastic bands, dumbbells); endurance; balance (walking on a straight line, walking heel to toe; ropes, sticks, balls, balloons); agility (challenging hand-eye coordination, foot-eye coordination, dynamic balance, standing and leaning balance, reaction time through ball games, relay races, dance movements and obstacle courses); stretching	Strength: 1 × 8–15 repetitions up to 3 × 8–15 repetitions	Strength: isokinetic knee extension, knee flexion, − left, − right, − 180°/s, − 60°/s (peak torque [Nm/kg]), isometric handgrip strength (force [N]); mobility: 30s chair stand (repetitions [number]), 8-ft up-and-go (time [s]); balance: 1-legged stance (time [s]); endurance: 6 min walking (distance [m])	*n* = 0; 72% (excluding drop-outs)	5
Morat & Mechling 2015 [15]	randomized, four-armed controlled trial	*n* = 78 (43/35); 68.4 ± 6.2 years; *n* = 26	EG1: multicomponent training (*n* = 20); EG2: resistance and balance training (*n* = 19); EG3: coordination training (*n* = 20); CG: no treatment (*n* = 19)	24 weeks; 2 sessions/week; 60 min/session	warm-up; strength (leg press, chest press, back extension, crunches, hip abduction and adduction; with strength training machines, gymnastic mats); balance (bipedal stance, single leg stance with soft pads, teeterboard); agility (stair climbing, uneven surfaces, avoiding obstacles, walking; with obstacles, stairs); cool-down	2 × 10–12 repetitions at 60–75% 1RM; 1 min rest between sets; 2 × 6–8 repetitions with 40–50% 1RM; 2 min rest between sets; intensity gradually increased by RPE	Strength: isometric leg press, chest press (force [N]), 1RM dynamic leg press, chest press (strength [kg]); mobility: MSOT, − maximal (time [s]), 5-times STS (time [s]), TUG (time [s]), maximum step length (distance [cm])	*n* = 0; IG1: 85%, IG2: 77%, IG3: 78%	6
de Resende Neto et al. 2020 [34]	randomized, two-armed controlled trial	*n* = 32 (0/32); n.a. ± n.a. years; *n* = 7	FT: multicomponent training (*n* = 16); TT: resistance and endurance training (*n* = 16)	12 weeks; 3 sessions/week; 55 min/session	Warm-up (main joint mobility, deadlifts, squats, jumps); agility/speed/coordination (up and down the step, rope training linear, medicine ball throws, displacement between cones, agility ladder, jumping jacks); strength/power (deadlift, rowing, sit and stand up, shoulder adduction, farmers walk, rowing, glute bridge, front plank; with suspension trainer, bench, kettlebells, elastic bands, medicine ball); endurance (high intensity interval running)	Agility/speed/coordination: 2 laps during circuit training; 1 min per exercise; 6–7/10 RPE; strength: 2 × 8–12 repetitions in 1 min; 7–9/10 RPE	Strength: 30s arm curl (repetitions [number]), maximal isometric dorsal strength ([kg]); mobility: 30s chair stand (repetitions [number]), rise and walk (time [s]); endurance: 6 min walking (distance [m])	n.a.; FT: 95%, TT: 85% (excluding drop-outs)	6
da Silva et al. 2019 [43]	randomized, two-armed controlled trial	*n* = 79 (n.a./n.a.); 68.8 ± 7.1 years; *n* = 35	Neuromotor: multicomponent (*n* = 40); aerobic: walking (*n* = 39)	12 weeks; 3 sessions/week; 50 min/session	Warm-up (stretching, mobilization, muscle activation, light calisthenic exercises); strength (light exercises with leggings, medicine ball, elastic resistance); balance and functional activities (functional gait training: circuit of obstacles; stationary march; ball throwing and kicking; standing up and sitting); muscle relaxation and breathing	Stretching: 30 s per segment; strength: 4 × 8 repetitions; 12–16 points on the Borg Scale of Perceived Exertion	Cognition: memory tests of perception (score), nomination (score), incidental memory (score), short-term memory (score), long-term memory (score), recognition (score)	n.a.; > 75% for all participants	4
Sohng et al. 2003 [44]	randomized, two-armed controlled trial	*n* = 52 (n.a./n.a.); 75.7 ± n.a. years; *n* = 7	EG: multicomponent training (*n* = 26); CG: no treatment (*n* = 26)	8 weeks; 2 sessions/week; 40 + min/session	stretching; strength (seated); endurance; balance; coordination; breathing, relaxation; health education	Increased session duration from 40 min until final week	Strength: isometric knee extension, knee flexion, ankle extension, ankle flexion, − left, − right (strength [kg])	n.a.; n.a.	6
Vaughan et al. 2014 [17]	randomized, two-armed controlled trial	*n* = 49 (0/49); 68.9 ± 3.4 years; *n* = 1	EG: multicomponent training (*n* = 25); CG: no treatment (*n* = 24)	16 weeks; 2 sessions/week; 60 min/session	endurance (choreographed movements in a random order to music, marching, side steps, arm movement, directional changes); strength (squats, arm and leg curls, elastic bands rows, weighted bag drags, ball bouncing, flies, push-ups; with chairs, kettlebells, balls, elastic bands, mats); balance (1-legged stance, heel-toe walking, stand on foam, step on foam); coordination/agility/reaction (weaving in and out of chairs, flat foot heel drumming, walking ball bounces, moving foot sequences, fast foot tapping, wall ball throws, catch dropping objects); stretching (back extension, cat and camel stretches, hamstring stretches, spinal rotation); cool-down	Endurance: 124–126 bpm (music) with 3–4/10 RPE to 126–128 bpm with 5–6/10 RPE; increasing number of simultaneous movements; strength: 2 × 6–8 repetitions with light weights to 5–6/10 RPE and 2x40sec; increasing weights as able; balance: 2x30sec to 2x40sec; increasing challenges to concentration; induced perturbation; reduced base of support; flexibility: 3–4/10 RPE; reaction: 2x30sec to 2x40sec; coordination/agility/reaction: smaller balls; faster movements	Mobility: TUG (time [s]); balance: 1-legged stance (n.a.); cognition: COAST (time [s]), COWAT (score), LNS (score), trail making test A/B (time [s]), Deary-Liewald reaction time task - simple reaction, − choice reaction (time [ms]); endurance: 6 min walking (distance [m])	n.a.; 94%	8
Wolf et al. 2020 [36]	randomized, two-armed controlled trial	*n* = 41 (0/30); n.a. ± n.a. years; *n* = 11	MG: multicomponent training (*n* = 12); SG: strength training (*n* = 18)	12 weeks, 3 sessions/week; 60 min/session	Warm-up; gait (rapid changes in movement direction); strength (lower limb exercises using body weight and/or elastic bands; balance (static and dynamic exercises); endurance (walking); stretching	Gait: progression was based on the complexity of task; strength: 3 × 12 repetitions with 50s rest, intensity based on progressively changing resistance of elastic bands; balance: progression based on augmenting the instability of the supporting surface; endurance: 12–14/20 RPE	Strength: maximal voluntary isometric contraction of hip, knee and ankle extensors and flexors (peak torque [Nm], rate of torque development [Nm/s]); balance: voluntary step execution test (time [s]); endurance: 6 min walking (distance [m]); mobility: 8-ft up-and-go (time [s]), 30s chair stand (repetitions [number]); gait: 8 m walking (speed [m/s])	n.a.; n.a.	5

*1RM* one repetition maximum, *bpm* beats per minute, *CDT* Clock Drawing Test, *COAST* California Older Adult Stroop Test, *COP* centre of pressure, *COWAT* Controlled Oral Word Association Test; *LNS* Letter-Number Sequencing Test, *M* mean, *MoCA* Montreal Cognitive Assessment, *MSOT* Multi-surface Obstacle Test for Older Adults, *n.a.* not available, *PEDro* Physiotherapy Evidence Database, *RPE* rate of perceived exertion, *SD* standard deviation, *STS* Sit to Stand Test, *TUG* Timed Up and Go Test, *LNS *Letter-Number Sequencing Test

Fifteen out of 20 studies used a two-armed [14, 16, 17, 19, 31, 34–40, 42–44], three studies a three-armed [18, 29, 40, 41] and two studies a four-armed study design [13, 15]. As it was part of the inclusion criteria, all studies applied standard randomization procedures for group assignment. According to the PEDro score, the median of the study quality was 6 and ranged from 4 [31, 37, 43] to 8 [17, 18, 35] (see Table 3). None of the studies blinded participants or therapists, since blinding is problematic within exercise intervention studies. Nine of the 20 included studies blinded the assessors [15, 17–19, 34–36, 41, 44]. Only two did not specifically report participant eligibility [18, 37].

**Table 3:** PEDro criteria and sum scores of the included studies

Study	Eligi-bility specified	Partici-pants randomly allocated	Concealed allocation	Similar baseline values	Blinding of participants	Blinding of therapist	Blinding of assessor	Dropout < 15%	Received treatment as allocated	Statistical between-group comparison	Point measures and variability provided	Sum (items 2 to 11)
Ansai et al. 2016 [29, 30]	√	√	√	√	–	–	–	√	√	√	√	7
Bohrer et al. 2019 [19]	√	√	–	√	–	–	√	–	–	√	√	5
Braga et al. 2020 [37]	–	√	–	√	–	–	–	–	–	√	√	4
Carvalho et al. 2009 [38]	√	√	–	√	–	–	–	√	–	√	√	5
Caserotti et al. 2008 [39]	√	√	–	√	–	–	–	√	–	√	√	5
Cwirlej-Sozanska et al. 2018 [13]	√	√	√	√	–	–	–	√	–	√	√	6
Englund et al. 2005 [40]	√	√	–	√	–	–	–	√	–	√	√	5
Freiberger et al. 2007 [41]	√	√	–	√	–	–	√	√	√	√	√	7
Karinkanta et al. 2007 [14]	√	√	√	√	–	–	–	√	√	√	√	7
Klusmann et al. 2010 [18]	–	√	√	√	–	–	√	√	√	√	√	8
Kovacs et al. 2013 [35]	√	√	√	√	–	–	√	√	√	√	√	8
Leite et al. 2015 [42]	√	√	√	√	–	–	–	–	–	√	√	5
Lord et al. 1996 [31, 32]	√	√	–	√	–	–	–	–	–	√	√	4
Marques et al. 2011 [16]	√	√	√	√	–	–	–	–	–	√	√	5
Morat & Mechling 2015 [15]	√	√	–	√	–	–	√	–	√	√	√	6
de Resende Neto et al. 2020 [34]	√	√	√	√	–	–	√	–	–	√	√	6
da Silva et al. 2019 [43]	√	√	–	√	–	–	–	–	–	√	√	4
Sohng et al. 2003 [44]	√	√	–	√	–	–	√	√	–	√	√	6
Vaughan et al. 2014 [17]	√	√	√	√	–	–	√	√	√	√	√	8
Wolf et al. 2020 [36]	√	√	–	√	–	–	√	–	–	√	√	5

PEDro scale obtained from the Physiotherapy Evidence Database [21]. Only the last ten items are summed up to the final score.

Although only four studies included cognitive measures, the outcome domains varied strongly (e.g. orientation, memory, language, attention, executive function, inhibition and more). Since an analysis in which all domains would be pooled would be too heterogeneous, it was decided to leave out cognition in further analyses.

### Risk of bias assessment

**Fig. 2:**
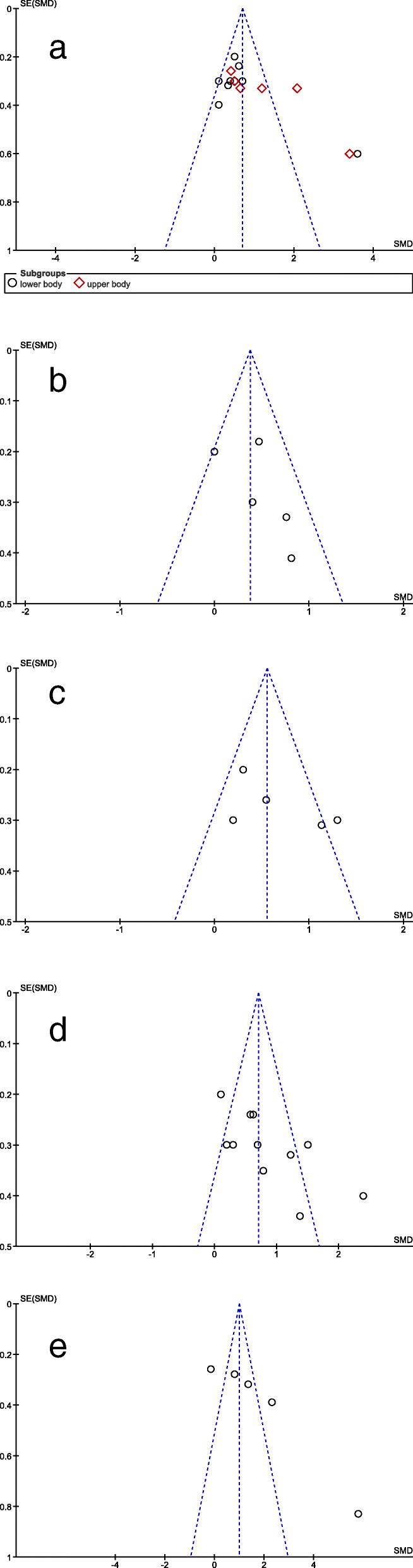
Funnel plots for bias assessment MAT vs. IC: **a** overall strength; **b** gait; **c** balance; **d**. mobility; **e** endurance. MAT = multimodal agility-like exercise training; IC = inactive control group; SE = standard error; SMD = standardized mean difference. The dashed line indicates the mean SMD

Funnel plots for all outcomes for the comparison of MAT vs. IC are shown in Fig. 2. They do not show a clear funnel-shape. This is also true for the separate plots of upper and lower body strength that are displayed together as overall strength. No studies with smaller sample sizes (higher standard errors) that usually scatter more widely at the bottom are present. The distribution of studies on the left and right side of the dashed standardized mean difference (SMD) line is equal for outcome measures of gait and balance, but not for strength, mobility and endurance.

### Data analyses of MAT vs. IC

**Fig. 3:**
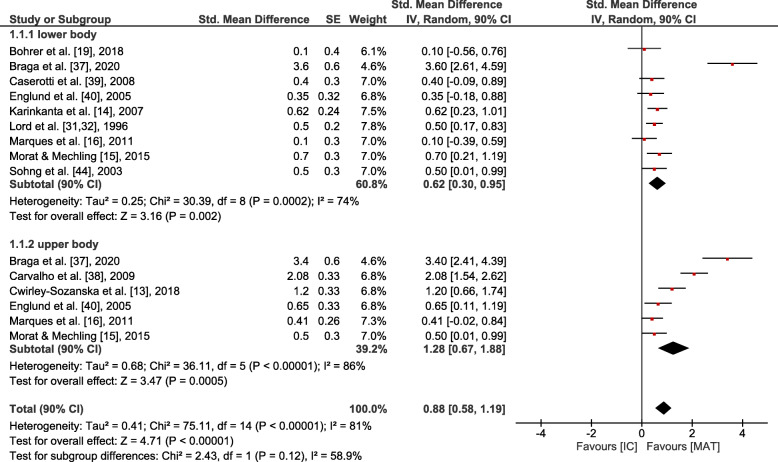
Outcomes of strength for MAT vs. IC. MAT = multimodal agility-like exercise training; IC = inactive control group; SE = standard error; IV = independent variable; CI = confidence interval

**Fig. 4:**
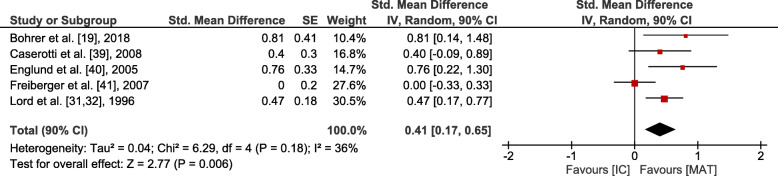
Outcomes of gait for MAT vs. IC. MAT = multimodal agility-like exercise training; IC = inactive control group; SE = standard error; IV = independent variable; CI = confidence interval

**Fig. 5:**
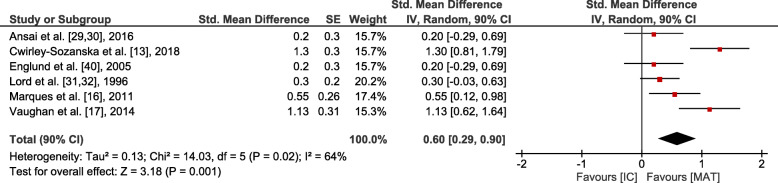
Outcomes of balance for MAT vs. IC. MAT = multimodal agility-like exercise training; IC = inactive control group; SE = standard error; IV = independent variable; CI = confidence interval

**Fig. 6:**
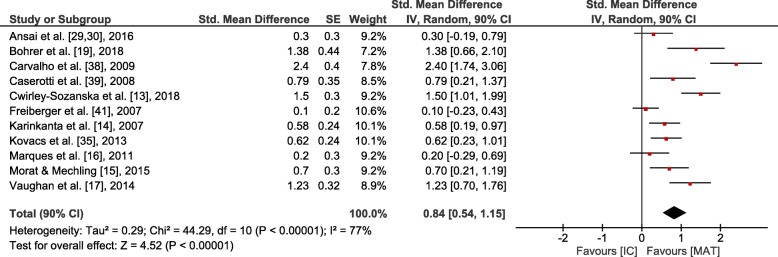
Outcomes of mobility for MAT vs. IC. MAT = multimodal agility-like exercise training; IC = inactive control group; SE = standard error; IV = independent variable; CI = confidence interval

**Fig. 7:**
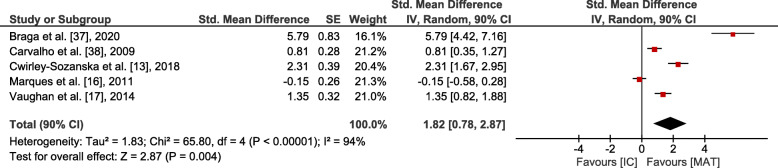
Outcomes of endurance for MAT vs. IC. MAT = multimodal agility-like exercise training; IC = inactive control group; SE = standard error; IV = independent variable; CI = confidence interval

Small overall effects with very low heterogeneity were observed in favour of MAT for gait (*p* = 0.006, SMD: 0.41 (90% CI: 0.17, 0.65), I^2^ = 0.36). Lower body strength (*p* = 0.002, SMD: 0.62 (90% CI: 0.3, 0.95), I^2^ = 0.74) and balance (*p* = 0.001, SMD: 0.6 (90% CI: 0.29, 0.9), I^2^ = 0.64) showed moderate effects at moderate to high heterogeneity. Upper body strength (*p* ≤ 0.001, SMD: 1.28 (90% CI: 0.67, 1.88), I^2^ = 0.86), overall strength (*p* < 0.001, SMD: 0.88 (90% CI: 0.58, 1.19), I^2^ = 0.81), mobility (*p* < 0.001, SMD: 0.84 (90% CI: 0.54, 1.15), I^2^ = 0.77) and endurance (*p* = 0.004, SMD: 1.82 (90% CI: 0.78, 2.87), I^2^ = 0.94) revealed large overall effects at large heterogeneity. However, all effects were significantly in favour of MAT (see Figs. 3, 4, 5, 6 and 7).

### Data analyses of MAT vs. AC

Few data were available for the comparison of effects of MAT vs. AC. No statistically significant effects were observed for any of the target outcome measures. Effects were mostly negligible, except for small effects on mobility and endurance in favour of MAT with large study heterogeneity (see Additional File 1 - Fig. S1 – Fig. S5): lower body strength (n (studies) = 4 [13, 15, 34, 36], *p* = 0.78, SMD: -0.05 (90% CI: − 0.32, 0.23), I^2^ = 0.11), upper body strength (n (studies) = 3 [15, 34, 42], *p* = 0.83, SMD: -0.04 (90% CI: − 0.36, 0.27), I^2^ = 0.00), overall strength (n (studies) = 5 [13, 15, 34, 36, 42], *p* = 0.92, SMD: -0.01 (90% CI: − 0.21, 0.19), I^2^ = 0.0), gait (n (studies) = 3 [36, 41, 42], *p* = 0.92, SMD: -0.02 (90% CI: − 0.34, 0.39), I^2^ = 0.46), balance (n (studies) = 2 [29, 36], *p* = 0.24, SMD: 0.41 (90% CI: − 0.17, 0.99), I^2^ = 0.52), mobility (n (studies) = 7 [13, 15, 29, 34, 36, 41, 42], *p* = 0.09, SMD: 0.25 (90% CI: 0.01, 0.48), I^2^ = 0.41), endurance (n (studies) = 3 [34, 36, 42], *p* = 0.72, SMD: 0.16 (90% CI: − 0.57, 0.89), I^2^ = 0.76).

## Discussion

To the best of our knowledge, no previously published meta-analytical review quantitatively evaluated the effects of multimodal agility-like exercise training (MAT) for community-dwelling older adults. The aim of this meta-analysis was to assess whether MAT provides superior effects compared to an inactive (IC) or alternative training control (AC) condition on physical and cognitive performance of older adults. According to the previously published agility framework by Donath et al. [12], we focus on multimodal agility-like training, where a combination of at least two traditional training domains (strength, balance, endurance) plus mandatory agility-like exercises (coordination and change of direction and velocity) are required, even if different terminology is used, and investigated effects on physical and cognitive performance. We found interventions ranging from a minimum of three domains (e.g. strength, balance, agility inspired exercises [15]) to five domains (e.g. flexibility, agility inspired exercises, coordination, strength, endurance [34]) but all following the multimodal agility-like rational. In other reviews on multimodal training, the content of the MT interventions was manifold and heterogeneous sometimes without clear categorisation framework and inclusion/ exclusion criteria were partly lacking [45–48]. Only one meta-analysis considered and specifically named “agility” as a potential training domain [48]. The characteristics of the interventions in our meta-analysis varied considerably with intervention durations ranging from 8 to 48 weeks (mean: 21.7 ± 11.9 weeks), two to three sessions per week (mean: 24.4 ± 0.5 total sessions) and a session duration of 40 to 90 min (mean: 58 ± 11.6 min), which is consistent with other analyses on MT [47]. Study samples in our meta-analysis were homogenous due to inclusion and exclusion criteria with most studies including participants with a mean age between 67 and 75 years. Only small sample sizes were missing, revealing a potential risk of bias. Outcome measures were manifold, like in other meta-analyses on MT [45–47] and were therefore grouped as strength, gait, balance, mobility, endurance and cognition. Within each domain, different target outcomes were pooled, which were mostly homogenous, except for strength outcomes with a variety of tests.

We found notable effects in favour of MAT compared to IC in all examined measures of physical performance. The largest effects in favour of MAT compared to IC were observed for measures of upper body strength, mobility and endurance. When comparing the effects of MAT vs. AC, effects were all insignificant and effect sizes were mostly negligible for physical performance. With large heterogeneity and few study comparison, small effects in favour of MAT compared to AC were observed for balance and mobility.

### Effects on strength

Effects of MAT on overall strength compared to an IC were large with moderate effects on lower body strength and large effects on upper body strength. In most of all included studies, strength training within MAT implied whole-body training with body weight or small devices (e.g. elastic bands, dumbbells). In all studies that examined the effects on strength in comparison to an AC, control groups had a focus on strength training. Still, AC and MAT did reveal similar effects on strength, whereas MAT is more likely to additionally induce improvements in other physical domains. For lower and upper body strength, the lowest effect sizes were observed for studies with limited study quality [16, 19]. Outcome measures of upper body strength were heterogeneous: studies that applied push-ups or arm curls revealed extremely large effect sizes [14, 37, 38], whereas moderate effect sizes were found in studies measuring isometric grip strength [38, 40] and isometric and dynamic chest press [15]. Higher effects on upper body strength compared to lower body strength might be due to higher similarities of testing and training.

Falck et al. [45] reviewed 48 studies of diverse exercise training regimen in older adults aged 60 years and also recommend MT for the improvement of strength performance. In 2004, Moreland et al. [49] claimed that muscle strength is a main aspect of fall prevention. Additionally, Granacher et al. [50] stated in their review that the effects of most strength training programs that are focusing on lower extremities are poorly translated into positive effects on balance, functional tasks, activities of daily living (ADL) and fall rates. Strength training as part of integrative MAT seems promising in terms of improvements of upper and lower body strength and may account for these translations.

### Effects on gait

As walking was included in MAT concepts of all but one studies [41], measures of gait showed effects in favour of MAT compared to an IC. Altogether, the included studies were relatively homogenous (I^2^ = 0.36) with similar underlying testing procedures. MAT is likely to improve habitual, as well as maximum gait speed. Mortaza et al. [51] showed that older adults who are categorized as fallers have a tendency towards slower gait speed and lower cadence, longer stride time and longer double support time. Regarding effects on different aspects of gait, the selection of suitable and sensitive gait analysis and target measures is decisive to assess effects of training programs on fall relevant aspects of gait. For the comparison to an AC, three studies showed no difference of effects on gait between groups.

### Effects on balance

Six studies with a moderately large heterogeneity (I^2^ = 0.64) were included in the analysis of effects on balance performance for MAT compared to IC with moderate effects in favour of MAT. Only two studies included balance measures with a comparison to an AC leaving insufficient data for analysis. All studies within the comparison to IC employed static balance conditions in testing and performed balance training in MAT as a combination of static and dynamic exercises in training. MAT might have even larger effects on dynamic balance that were only assessed in one of the included studies. Only Cwirlej-Sozanska et at [13]. also included functional outcomes (functional reach, tandem walk, tandem pivot). Since MAT emphasizes dynamic balance tasks, such as agility and coordination, this more functional testing approach might account for larger effects in this study, according to the task-specificity principle of neuromuscular training adaptations, which is especially relevant for balance training [4]. Functional balance, allowing for movement patterns in time and space without losing balance, are crucial for older adults’ daily activities and thus should be part of MAT [12]. With its variety of training domains, MAT can easily include dynamic and functional balance demands in a specific balance tasks or embedded in agility tasks. Vaughan et al. [17], who reported the second highest effect size in favour of MAT compared to IC, were among the few studies that reported progression of balance training.

### Effects on mobility

Large effects were observed for mobility outcomes in favour of MAT compared to an IC. There were relevant but small effects in comparison to an AC in favour of MAT but without statistical significance. Since the terminology “agility” for older adults is neither established nor used much in literature, “agility” was comprehensively understood as coordination and change of direction and velocity in our meta-analysis. Thus, exercises for agility, also addressing mobility outcomes, were present in all studies but appeared differently. The most frequently used mobility measure was the TUG and the STS test. Carvalho et al. [38] revealed extremely large effects of MAT on mobility compared to IC. It strikes out that their MAT design incorporated some aspects of the original agility concept by Donath et al. [12] as they included at least one of the following criteria: change of direction, change of velocity (start stop), balance (static and dynamic), strength and endurance in MAT in different forms and not always all aspects of agility.

Among the studies with the highest SMD for mobility were two that simultaneously revealed the highest effects on balance outcomes [14, 17] and on gait [19]. Dynamic aspects of balance and improvements in gait might show transfer effects on mobility measures like the Timed Up and Go Test for older adults, where participants must walk and turn, maintaining an upright posture. However, a relatively high methodological heterogeneity of studies makes it hard to draw a further conclusion. The same is true for the comparison of effects of MAT on mobility compared to an AC. Small but insignificant effects tend to show that MAT is a better means to improve mobility and therefore functional abilities in older adults as compared to resistance training alone or combined with one additional training domain only. MAT should imply agility-specific exercises involving changes of direction and velocity, balance, strength, and endurance components, following the agility approach by Donath et al. [12]. This seems particularly promising for everyday life activities for older adults in which they also must turn, accelerate, decelerate, and stand without losing balance and without fatiguing. Other meta-analyses, investigating effects of MT also report greater effects of MT on mobility outcomes compared to IC as well as AC [45–47]. However, the perceptual aspects of agility training and the characteristics of an original definition by Sheppard and Young [52]: “a rapid whole-body movement with change of velocity or direction in response to a stimulus”, comprising a perceptual decision-making process and its outcome, a change of direction or velocity task is often missing in recent studies. A recently published pilot study [53] specifically included agility-based exercises being based upon the agility approach by Donath et al. [12]. In addition, Morat et al. [54] published the protocol of their planned RCT evaluating agility training for older adults.

### Effects on endurance

Despite a high heterogeneity of studies, the effects of MAT on endurance were large compared to IC. All but one studies on endurance performance included a specific endurance exercises domain within MAT, whereas a detailed training prescription was only provided in few studies. All except one study applied the 6 min walking test as an outcome measure for endurance. Cwirley-Sozanska et al. [13] performed the 2 min step test revealing large effects. The 2 min step test appears to benefit from other physical domains like strength and balance besides endurance exercises and agility-specific tasks might additionally address more anaerobic endurance. Marques et al. [16] showed negligible effects in favour of IC (SMD = − 0.15) although they included exercises like marching, stepping, ball games and relay races in MAT that seem to beneficially affect endurance. A more anaerobe endurance test (like the 2 min step test) might have led to different results. Effects of MAT on endurance performance can be expected by integrating classical endurance components, but also by planning and progressing other training domains like agility or coordination in a way that a cardiovascular stimulus is induced. This is in line with results of a meta-analysis that reviewed effects of MT in older populations compared to IC and AC and revealed greater effects on peak oxygen consumption of MT compared to an endurance training AC and IC [46].

### Effects on cognition

As it was previously described, cognition was left out of any further analysis due to the heterogeneity of outcome domains within the four relevant studies. But it seems worth investigating, if adding cognitive demands to MAT exercises would enlarge effects on cognitive performance measures [22].

### Limitations

This meta-analysis is the first that evaluates the effects of MAT in older adults with pooled effect sizes for the comparison to AC and IC and was reported according to the PRISMA guidelines [20]. One limitation that needs to be mentioned is that risk of bias assessment indicated potential bias from missing studies with small sample sizes. Also, due to a lack of studies including similar testing procedures, pooling of several testing procedures for some outcome measures was performed which compromises the significance of findings. Study heterogeneity varied between I^2^ = 0.0 and I^2^ = 0.94 between outcome measures. Despite noteworthy heterogeneity concerning sample sizes, intervention duration and study arms, the findings offer a unique comprehensive qualitative view on recent scientific evidence on the effects of multimodal agility-like exercise interventions with a convenient pool of data. Effects of MAT vs. AC might be more biased because of the heterogeneity of control conditions. Additionally, the low number of studies that included AC requires more studies with multiple study arms and high study quality. Within our meta-analysis, no specific health and fall prevention outcomes have been considered, however, they could indirectly benefit from agility exercises.

## Conclusions

Multimodal agility-like exercise training (MAT) can improve different physical performance aspects relevant for healthy and successful aging of older adults. The effects were comparable to those of alternative exercise training regimen. Thus, this meta-analysis showed that MAT might offer a time-efficient training option for older adults, since positive effects in many measures of physical performance can be achieved with a training volume that traditionally just allows for the training of one or two physical domains. Studies partly include selective aspects of MAT but lack clear definitions or categorization towards the agility framework by Donath et al. [12]. According to this framework, exercises comprising changes of direction and velocity, as well as exercises for improving balance, strength and endurance are essential to train relevant abilities for everyday life activities of older adults. Comprehensive multimodal agility training concepts bring the advantage of reproducing real-life conditions and therefor offer more opportunity for transfer; however, this must be investigated more. With this in mind, the results of a first pilot-study [53] are promising and the evaluation of an innovative agility training approach within an RCT study [54] could provide further insights about the effects of agility training in older adults. Thus, the present meta-analysis highlights the importance of MAT for older adults and provides important insights for future training conceptualization. The systematic application of exercise science principles and load control during agility training in long-term intervention studies is required.

## Supplementary Information

**Additional file 1.**

## Data Availability

Data sharing is not applicable to this meta-analysis as no datasets were generated or analysed. All data generated or analysed are included in this published article [and its supplementary information files].

## Abbreviations

AC: Active controls
ADL: Activities of daily living
CI: Confidence intervals
IC: Inactive controls
MAT: Multimodal agility-like exercise training
MT: Multimodal exercise training
PED: Physiotherapy Evidence Database
RCT: Randomized controlled trial
SMD: Standardized mean differences
STS: Sit to Stand Test
TUG: Timed Up and Go Test
