# Supplementary material for: Effects of multimodal agility-like exercise training compared to inactive controls and alternative training on physical performance in older adults: a systematic review and meta-analysis
Source: Eur Rev Aging Phys Act. 2021 Feb 25;18:4. doi: 10.1186/s11556-021-00256-y (PMC7908670; doi:10.1186/s11556-021-00256-y)
Supplement: Supplementary file 1 — Additional file 1. [file 11556_2021_256_MOESM1_ESM.pdf]

Study or Subgroup	Std. Mean Difference	SE	Weight	Std. Mean Difference IV, Random, 90% CI
**2.1.1 lower body**				
de Resende Neto et al. [34], 2020	0.6	0.41	8.6%	0.60 [-0.07, 1.27]
Karinkanta et al. [14], 2007	-0.11	0.23	27.3%	-0.11 [-0.49, 0.27]
Morat & Mechling [15], 2015	-0.1	0.3	16.0%	-0.10 [-0.59, 0.39]
Wolf et al. [36], 2020	-0.4	0.4	9.0%	-0.40 [-1.06, 0.26]
**Subtotal (90% CI)**			**60.9%**	**-0.05 [-0.32, 0.23]**
Heterogeneity: Tau^2 = 0.01; Chi^2 = 3.37, df = 3 (P = 0.34); I^2 = 11%				
Test for overall effect: Z = 0.27 (P = 0.78)				
**2.1.2 upper body**				
de Resende Neto et al. [34], 2020	0.39	0.4	9.0%	0.39 [-0.27, 1.05]
Leite et al. [42], 2015	0	0.32	14.1%	0.00 [-0.53, 0.53]
Morat & Mechling [15], 2015	-0.1	0.3	16.0%	-0.10 [-0.59, 0.39]
**Subtotal (90% CI)**			**39.1%**	**0.05 [-0.27, 0.36]**
Heterogeneity: Tau^2 = 0.00; Chi^2 = 1.00, df = 2 (P = 0.61); I^2 = 0%				
Test for overall effect: Z = 0.25 (P = 0.80)				
**Total (90% CI)**			**100.0%**	**-0.01 [-0.21, 0.19]**
Heterogeneity: Tau^2 = 0.00; Chi^2 = 4.53, df = 6 (P = 0.60); I^2 = 0%				
Test for overall effect: Z = 0.10 (P = 0.92)				
Test for subgroup differences: Chi^2 = 0.14, df = 1 (P = 0.71), I^2 = 0%				

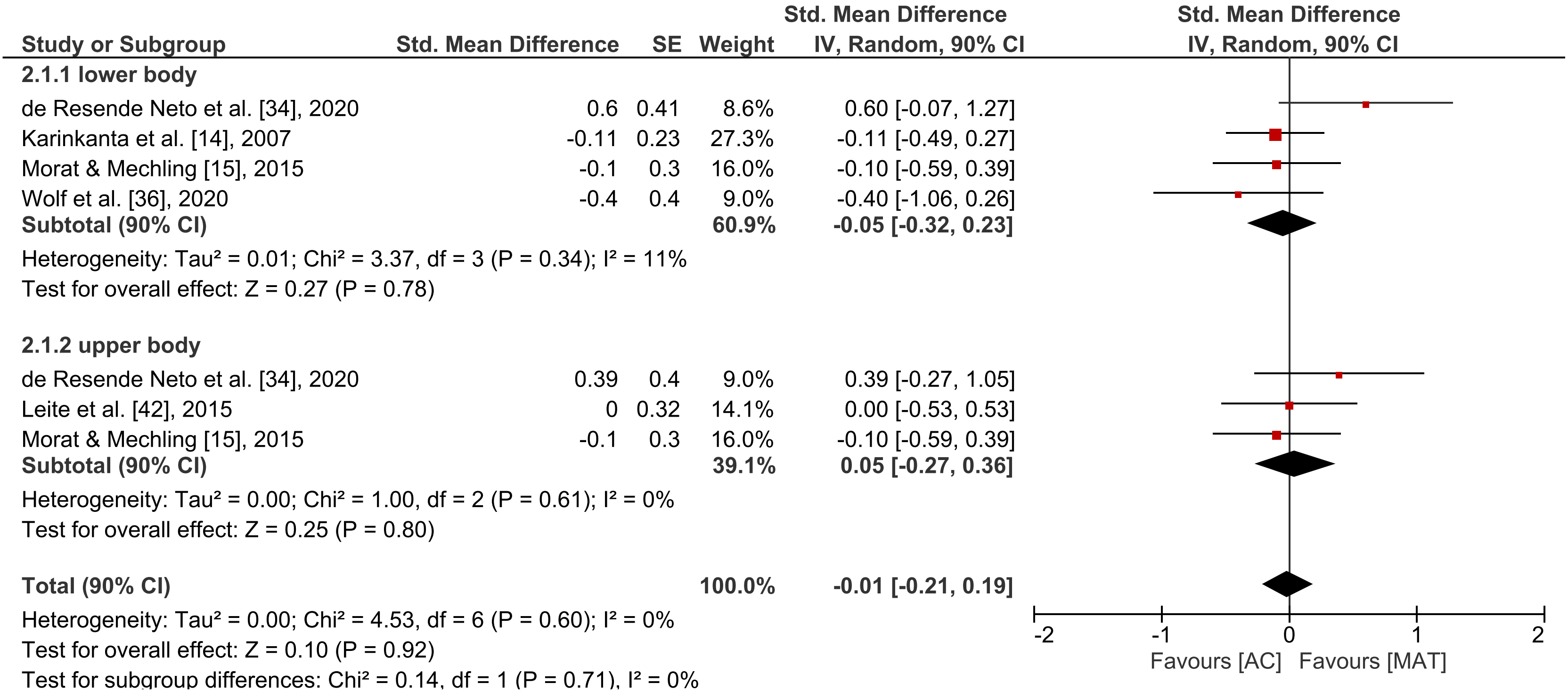

Supplementary Figure S1. Outcomes of strength for MAT vs. AC; MAT = multimodal agility-like exercise training; AC = active control group; SE = standard error; IV = independent variable; CI = confidence interval.

Study or Subgroup	Std. Mean Difference	SE	Weight	Std. Mean Difference IV, Random, 90% CI	Std. Mean Difference IV, Random, 90% CI
Freiberger et al. [41], 2007	-0.2	0.2	45.5%	-0.20 [-0.53, 0.13]	
Leite et al. [42], 2015	-0.1	0.3	31.2%	-0.10 [-0.59, 0.39]	
Wolf et al. [36], 2020	0.62	0.38	23.3%	0.62 [-0.01, 1.25]	
**Total (90% CI)**			**100.0%**	**0.02 [-0.34, 0.39]**	

Heterogeneity: Tau² = 0.07; Chi² = 3.70, df = 2 (P = 0.16); I² = 46%
Test for overall effect: Z = 0.10 (P = 0.92)

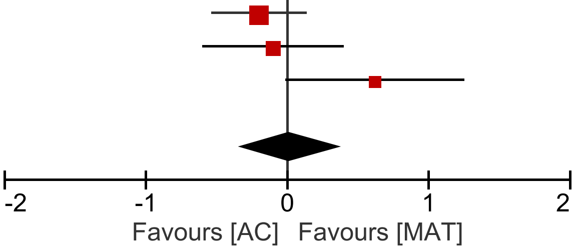

Supplementary Figure S2. Outcomes of gait for MAT vs. AC; MAT = multimodal agility-like exercise training; AC = active control group; SE = standard error; IV = independent variable; CI = confidence interval.

Study or Subgroup	Std. Mean Difference	SE	Weight	Std. Mean Difference IV, Random, 90% CI
Ansai et al. [29,30], 2016	0.1	0.3	56.2%	0.10 [-0.39, 0.59]
Wolf et al. [36], 2020	0.81	0.39	43.8%	0.81 [0.17, 1.45]
**Total (90% CI)**			**100.0%**	**0.41 [-0.17, 0.99]**

Heterogeneity: Tau² = 0.13; Chi² = 2.08, df = 1 (P = 0.15); I² = 52%
Test for overall effect: Z = 1.17 (P = 0.24)

Std. Mean Difference
IV, Random, 90% CI

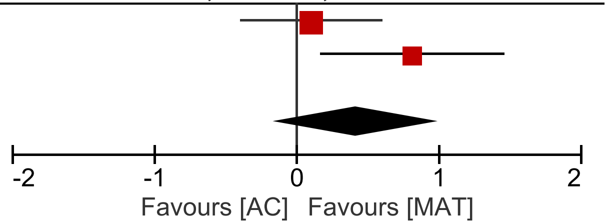

Supplementary Figure S3. Outcomes of balance for MAT vs. AC; MAT = multimodal agility-like exercise training; AC = active control group; SE = standard error; IV = independent variable; CI = confidence interval.

Study or Subgroup	Std. Mean Difference	SE	Weight	Std. Mean Difference IV, Random, 90% CI	Std. Mean Difference IV, Random, 90% CI
Ansai et al. [29,30], 2016	0.1	0.3	14.0%	0.10 [-0.39, 0.59]	
de Resende Neto et al. [34], 2020	1	0.4	9.5%	1.00 [0.34, 1.66]	
Freiberger et al. [41], 2007	-0.2	0.2	21.2%	-0.20 [-0.53, 0.13]	
Karinkanta et al. [14], 2007	0.31	0.24	18.0%	0.31 [-0.08, 0.70]	
Leite et al. [42], 2015	0.1	0.32	13.0%	0.10 [-0.43, 0.63]	
Morat & Mechling [15], 2015	0.6	0.3	14.0%	0.60 [0.11, 1.09]	
Wolf et al. [36], 2020	0.26	0.38	10.3%	0.26 [-0.37, 0.89]	
**Total (90% CI)**			**100.0%**	**0.25 [0.01, 0.48]**	

Heterogeneity: Tau² = 0.06; Chi² = 10.22, df = 6 (P = 0.12); I² = 41%
Test for overall effect: Z = 1.71 (P = 0.09)

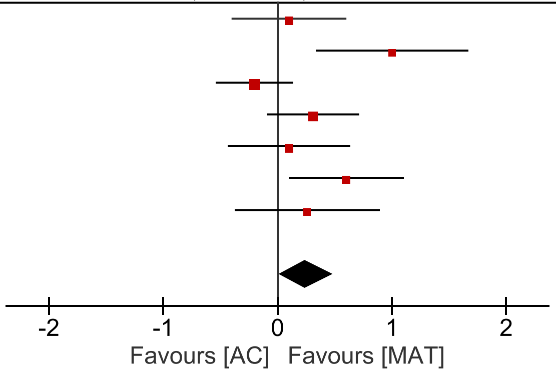

Supplementary Figure S4. Outcomes of mobility for MAT vs. AC; MAT = multimodal agility-like exercise training; AC = active control group; SE = standard error; IV = independent variable; CI = confidence interval.

Study or Subgroup	Std. Mean Difference	SE	Weight	Std. Mean Difference IV, Random, 90% CI
de Resende Neto et al. [34], 2020	0.86	0.42	31.5%	0.86 [0.17, 1.55]
Leite et al. [42], 2015	-0.62	0.33	35.3%	-0.62 [-1.16, -0.08]
Wolf et al. [36], 2020	0.33	0.38	33.2%	0.33 [-0.30, 0.96]
**Total (90% CI)**			**100.0%**	**0.16 [-0.57, 0.89]**

Heterogeneity: Tau² = 0.45; Chi² = 8.38, df = 2 (P = 0.02); I² = 76%

Test for overall effect: Z = 0.36 (P = 0.72)

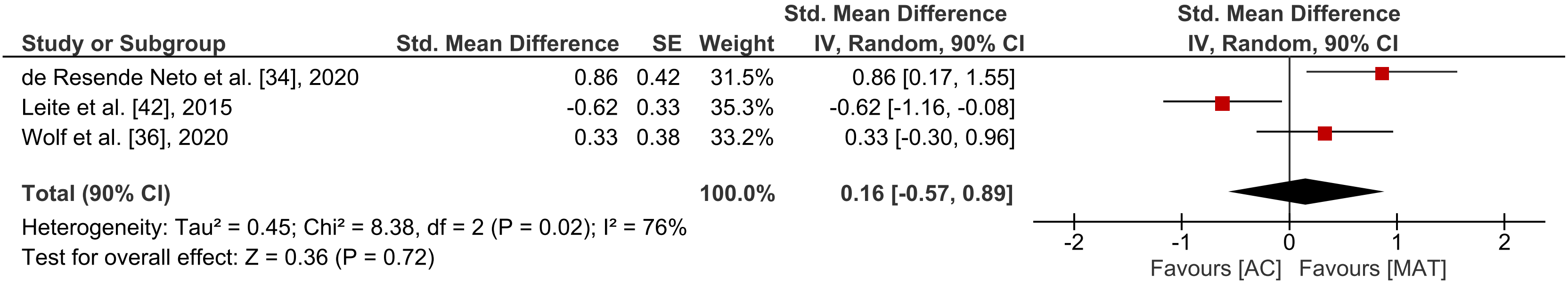

Supplementary Figure S5. Outcomes of endurance for MAT vs. AC; MAT = multimodal agility-like exercise training; AC = active control group; SE = standard error; IV = independent variable; CI = confidence interval.
